# *Phaseolus coccineus* L. Landraces in Greece: Microsatellite Genotyping and Molecular Characterization for Landrace Authenticity and Discrimination

**DOI:** 10.3390/biotech13020018

**Published:** 2024-06-07

**Authors:** Irene Bosmali, Georgios Lagiotis, Ioannis Ganopoulos, Eleni Stefanidou, Panagiotis Madesis, Costas G. Biliaderis

**Affiliations:** 1 Laboratory of Food Chemistry and Biochemistry, Department of Food Science and Technology, School of Agriculture, Aristotle University of Thessaloniki, University Campus, 54124 Thessaloniki, Greece; mposmali@agro.auth.gr; 2 Institute of Applied Biosciences, CERTH, 6th km Charilaou-Thermis, 57001 Thessaloniki, Greece; glagiotis@certh.gr (G.L.); elenuba@certh.gr (E.S.); 3 Laboratory of Molecular Biology of Plants, Department of Agriculture Crop Production and Rural Environment, University of Thessaly, Fytokou St., 38446 Volos, Greece; 4 Institute of Plant Breeding and Genetic Resources, ELGO-DIMITRA (ex NAGREF), 1st District Road of Thessalonikis-Polygyrou, 57001 Thermi, Greece; giannis.ganopoulos@gmail.com

**Keywords:** Gigantes, Elephantes, Prespon, capillary electrophoresis, EST-SSR, *P. vulgaris* L.

## Abstract

*Phaseolus coccineus* L. is a highly valuable crop for human consumption with a high protein content and other associated health benefits. Herein, 14 *P. coccineus* L. landraces were selected for genetic characterization: two Protected Geographical Indication (PGI) landraces from the Prespon area, namely “Gigantes” (“G”) and “Elephantes” (“E”), and 12 additional landraces from the Greek Gene Bank collection of beans (PC1–PC12). The genetic diversity among these landraces was assessed using capillary electrophoresis utilizing fluorescence-labeled Simple Sequence Repeat (SSR) and Expressed Sequence Tag (EST); Simple Sequence Repeat (SSR) is a molecular marker technology. The “G” and “E” Prespon landraces were clearly distinguished among them, as well as from the PC1 to PC12 landraces, indicating the unique genetic identity of the Prespon beans. Overall, the genetic characterization of the abundant Greek bean germplasm using molecular markers can aid in the genetic identification of “G” and “E” Prespon beans, thus preventing any form of fraudulent practices as well as supporting traceability management strategies for the identification of authenticity, and protection of the origin of local certified products.

## 1. Introduction

*P. coccineus* L. (runner bean) is an annual legume crop, commonly grown for its immature green pods and dry seeds, particularly in small- and medium-sized farming operations [1,2]. In the region of the “Mikri Prespa” lake in Greece, beans, and especially runner beans, are the only grown crops, significantly boosting the local economy [3,4]. The runner bean landraces “Elephantes” (“E”) and “Gigantes” (“G”), which have contrasting characteristics in terms of seed size and shape, are traditionally grown in the Prespes Lake region (NW Greece) and marketed as “dry beans” [5].

Traditional methods to characterize landraces of agricultural crops, such as the analysis of morphological characteristics, are more intrusive, time-consuming, and less precise since the morphological features and plant tissue composition can be greatly influenced by environmental conditions and agricultural practices [6]. Instead, molecular techniques provide more user-friendly and effective methodologies for the analysis of the genetic makeup and, thereby, the identification of crop plant species and varieties, including beans [7]. From an agronomic standpoint, allogamy and cold tolerance are further distinguishing traits of runner bean cultivation that have been significantly studied. Various molecular marker analyses, such as Random Amplification of Polymorphic DNA (RAPD), Amplified Fragment Length Polymorphism (AFLP), Restriction Fragment Length Polymorphism (RFLP), and Simple Sequence Repeat (SSR) [7], have been extensively utilized to explore the genetic diversity in populations of agricultural crops [8]. SSRs, specifically, are 2–6 bp short tandem repeats, codominant DNA markers that are extensively prevalent in plant genomes [9]. SSR marker analyses have been extensively applied to many plants, including *Phaseolus* species [10,11,12]. Expressed sequence tag-SSRs (EST-SSRs) are created by SSR mining in ESTs that are acquired by cDNA partial sequencing [13,14]. Between them, SSRs are found in libraries of genomic DNA and exhibit more polymorphisms than EST-SSR [15].

Despite the current decade’s genome sequencing of many legume species and the extensive development of molecular markers, very limited research has been conducted on runner beans from a genomic perspective [1], which further hinders targeted breeding attempts to improve the use and authentication of certain landraces of this species. Nevertheless, the characterization of the genetic diversity present amongst various *P. coccineus* L. accessions and the assessment of genetic bottlenecks have been achieved by the development and application of nuclear and chloroplast SSR markers [10,11,16,17]. Quite recently, the sequencing of the *P. coccineus* genome, as a part of a large sequencing study including several *Phaseolus* species, also revealed a significant genetic divergence in populations from *Phaseolus vulgaris* L. [18].

In northwestern Greece, the Prespes Lake area is well known for its bean production, with some dry legume grains being certified as PGI [5]. In order to safeguard these products from tampering and intentional adulteration, which has been an increasing problem in recent years, it is essential for the agricultural communities from this geographical region to be able to molecularly identify the different bean landraces and authenticate their regional products. In the present study, 15 Greek bean landraces were genetically characterized using fluorescent-labeled SSR and capillary electrophoresis. This technique has been widely employed in similar studies for many plant species [19,20,21]. Our analysis revealed the genetic variation present amongst the studied landraces and established an effective method for species/landrace identification and discrimination. This method can further support traceability management strategies for the identification of authenticity and the protection of the origin of local *P. coccineus* L. landraces.

## 2. Materials and Methods

### 2.1. Plant Material

**Figure 1:**
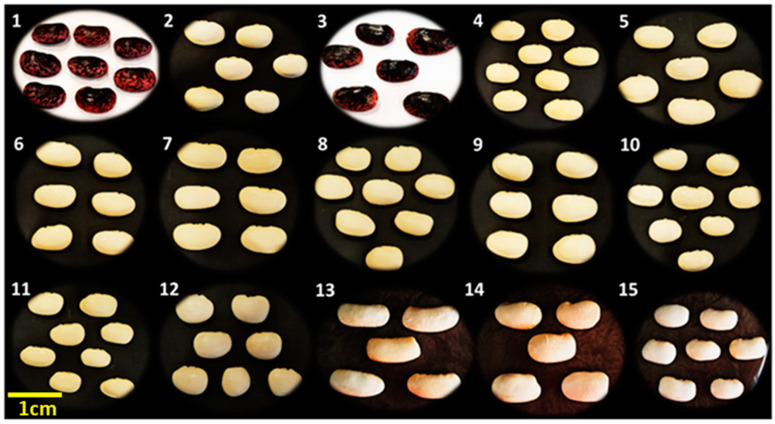
Dry beans (*P. coccineus* L. landraces) used in this study. For landrace coding, see Table 1 and Table 2.

**Figure 2:**
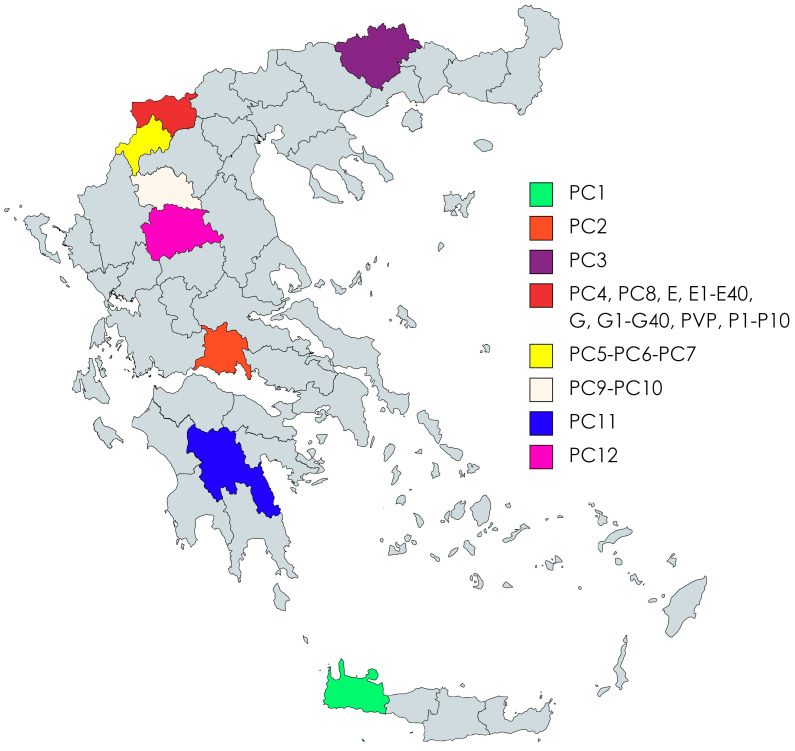
Geographic distribution of the bean landraces/varieties used in this study (For more information, see Table 1 and Table 2).

The local bean varieties were retrieved from collections at the Institute of Plant Breeding and Genetic Resources (ELGO ‘Demeter’, 57001, Thermi, Thessaloniki). The first set of 15 bean landraces comprised 12 Greek *P. coccineus* L. landraces from the Gene Bank, two *P. coccineus* L. landraces from the Prespes Lake area region, namely Gigantes “G” and Elephantes “E”, and one *P. vulgaris* L (PVP) Prespon variety (Table 1, Figure 1) as an outlier for comparison purposes. The second set included eight *P. coccineus* L. genotypes, four “G” and four “E” Prespon landraces, as well as one genotype “PVP” Prespon variety (Table 2, Figure 1), with ten biological replicates per genotype (Figure 2).

### 2.2. DNA Extraction and EST-SSR/SSR Analysis

For the molecular characterization, ten plants from each accession were chosen. Genomic DNA was isolated from young leaf tissue using the Doyle & Doyle method [22]. A UV–Vis Spectrophotometer Q5000 (Quawell Technology Inc., San Jose, CA, USA) and 1% agarose gel electrophoresis were used to evaluate DNA quantity and quality. All samples were diluted at 20 ng/μL for PCR.

PCR amplification was performed in a total volume of 20 μL, with 20 ng of template DNA, 10x PCR buffer, 200 μM of each dNTP, 10 pmol of each primer, and 1U Kapa Taq polymerase. Reactions were performed in a PCR Thermocycler (Sensoquest GmbH, Gottingen, Germany). A set of seven SSR and seven EST-SSR markers was utilized to authenticate and discriminate the *P. coccineus* L. landraces and the *P. vulgaris* L. variety from Greece (Table 3). However, the EST-SSR molecular markers used were designed by our team based on the RNA analysis performed earlier [5].

Optimal amplification conditions were determined by performing several test runs per marker. A rapid PCR protocol was performed using an initial denaturing step of 95 °C for 5 min, followed by 35 cycles of 95 °C for 5 s, 47–49 °C for 10 s, and 72 °C for 10 s, and then a final extension step of 72 °C for 2 min. The same protocol was also applied to the EST-SSR microsatellites.

### 2.3. Capillary Electrophoresis Detection

Using the FAM, HEX, TAM, and ROX fluorescent dyes, 14 pairs of fluorescent primers were produced (Table 3). Fluorescence-labeled SSR amplicons were analyzed with capillary electrophoresis to determine the fragments’ size and the position of the target peaks in relation to the internal standard in the same capillary lane. Using a continuous pipette, a 96-well reaction plate was filled with a mixture of highly deionized formamide (HIDI) and a LIZ600 molecular weight internal standard at a volume ratio of 27:1. Each well held a total volume of 15 μL, to which 1 μL of PCR amplification product, diluted 50 times, was added. The well plate was sealed with an adhesive plate film and was subjected to a 500× *g* centrifugation.

The denaturation process in the PCR machinery was carried out for five minutes at 95 °C without heating the hot cover. Upon completion of the operation, the 96-well plate was transferred to ice for 5 min. After cooling, the well plate was shortly centrifuged at 2000× *g*. An ABI 3500 series genetic analyzer (ABI Corporation, Foster City, CA, USA) was used to detect the samples. Geneious v5.0 (Geneious Prime^®^2019.0.4) was then used to analyze the initial data and compare each sample’s peak position with the molecular weight internal standard in each pool. This allowed for the determination of the fragment size.

### 2.4. Data Analysis

The results of the capillary electrophoresis analysis of the samples were used to generate the codominant data matrix. The matrices were analyzed using GenAlex ver. 6.5 software [23]. The Nei and Li/Dice similarity index [24] was used to determine how comparable the qualitative data were, and the Unweighted Pair Group Method and Arithmetic Averages (UPGMA) were used to examine the similarity estimations. The GenAlex-calculated mutual coefficients of similarity matrices were exported and examined, and the resulting clusters were represented as dendrograms using the MEGA X program [25].

Using the GenAlex ver. 6.5 software [23], an analysis of molecular variance (AMOVA) [26] was used to assess the hierarchical distribution of genetic variation both among and within landraces. A cluster analysis using a UPGMA dendrogram [27] was carried out by the software GenAlex ver. 6.5. The genetic structures of *P. coccineus* L. landraces were examined using principal coordinate analysis (PCoA) based on standardized covariance of genetic distance for codominant markers [28]. The standardized option, which is predicated on transforming the distance matrix into a covariance matrix, was adopted in this investigation. By utilizing this procedure, the square root of n-1 is divided by the relevant covariance input. The Nei’s standard genetic distance [24] between pairs of populations was computed at the population level for the codominant data set. Afterward, GenAlEx ver. 6.5 uses this geometric distance matrix to perform PCoA analysis on codominant data. The multivariate data set with many loci and different samples might be analyzed using PCoA to identify and visualize the key trends.

The MEGA X program [25] was used to translate the mutual coefficients of similarity matrices computed in GenAlEx ver. 6.5 by producing clusters that were depicted as dendrograms. A bootstrap analysis was used to evaluate the dendrograms’ robustness after 1000 iterations, with the analysis also being performed by GenAlEx ver. 6.5 and MEGA X. Genetic parameters (No of individuals (N), No. of Different Alleles (Na); Effective number of alleles per locus (Ne); Observed heterozygosity (Ho), Expected heterozygosity (He); Shannon’s information index (I); Percentage of polymorphic loci (P %); Gene differentiation coefficient (F_st_)) were determined by the GenAlEx software ver. 6.5.

### 2.5. Population Structure

To evaluate population structure, the STRUCTURE software version 2.3.1 [29] was used to provide a grouping of the 15 *Phaseolus* species landraces/varieties, using a Bayesian method (100,000 burn-ins and 100,000 Markov Chain Monte Carlo reps after burn-in) under the admixture model. The appropriate number of clusters (K) was determined according to Evanno et al. [30]. The logarithm posterior probability for each K was determined by testing three replicate runs per K value and a range of population sizes (K = 1 to 10). When the likelihood of a greater K peaked, the total number of populations was fixed. Using Evanno’s et al. [30] method, the best K value was found using the STRUCTURE HARVESTER online tool [31]. If the associated membership probability value of a landrace or variety was more than 0.8, it was classified into a specified cluster; if not, it was deemed to be an admixed population.

## 3. Results

### 3.1. Molecular Characterization of P. coccineus L. Landraces Based on SSR and EST-SSR Molecular Markers

To genetically characterize the 15 local Phaseolus species landraces/varieties, SSR and EST-SSR molecular markers were used. This section presents the results of their molecular characterization as PCoA analyses, UPGMA dendrograms, and STRUCTURE analyses.

Fourteen SSR and EST-SSR markers were tested on fourteen *P. coccineus* L. landraces around Greece, as well as a *P. vulgaris* L. variety. Seven of the SSR markers were previously developed for *P. vulgaris* L. and seven were further developed by our team for *P. coccineus* L. (Table 3). For DNA fingerprinting, a total of 15 polymorphic bands were obtained from the 14 pairs of SSR and EST-SSR primers (Table 3). An average of 6.2 alleles per locus were produced by the chosen SSR primers, with the range being from 3 (AZ301561) to 10 (AY298744). On average, 3.14 alleles were estimated per locus, with values ranging from 2 (AZ301561, AZ044945) to 5 (X80051). On the other hand, an average of 2.71 alleles per locus were detected for the analyzed EST-SSR markers, ranging from 1 (hyp and Sial) to 5 (ser and bHLH).

Seven of the primer pairs were monomorphic (EST-SSRs) for most plant samples of the overall *P. coccineus* L. landraces and the *P. vulgaris* L. variety (Table 1). Seven of the primers (50%) (SSRs) generated reproducible and clear polymorphic bands (Table 3). The majority of the observed band sizes matched the predicted size ranges (Table 3). On each of the ninety unique samples of “G” and “E” landraces, as well as a “PVP” Prespon variety collected from the Prespes Lake region in Greece, each of the fourteen pairs of primer was examined. Only one SSR marker (X 80051) and five EST-SSR primer pairs (catalase, hyp, transmemb, sial, and ser) exhibited monomorphic band patterns among the analyzed samples. The rest of the primer pairs were polymorphic.

As shown in Table 4, landraces from Agios Germanos Prespes (PC4) showed the highest levels of polymorphisms (P = 86.67%), followed by Kastoria (PC5), Lechovo (PC8), Grevena-Monaxiti (PC9), Arkadia (PC11), and Trikala (PC12); the lowest polymorphisms (P = 66.67%) were shown by Chania (PC1) and Fokida (PC2). The highest level of heterozygosity was shown by Arkadia (He = 0.407) and the lowest by Gigantes (He = 0.164), followed by Elephantes (He = 0.186), which is expected as the Gigantes and Elephantes Prespon are the products of a breeding process with selfing. Overall, the F_st_ was high (F_st_ = 0.435) as the populations studied in Table 4 are landraces that have not been the subject of a selection and breeding process, with the exception of “G”, “E”, and “PVP”, thus a high variance is expected.

Genetic variation estimates on species and location levels based on the markers are summarized in Table 4 and Table 5. Overall, low levels of genetic variation were observed at a variety level, with I = 0.099 and He = 0.071 for Elephantes and I = 0.297 and He = 0.214 for Gigantes, respectively. Among the studied landraces/varieties, Elephantes exhibited higher total polymorphisms (P = 40%) than Gigantes (P = 33.33%). Regarding F_st_ = 0.802, which is close to 1, the calculated value indicates that the examined populations have limited sharing of genetic material. This is expected as both groups of landraces, although originating from a mixed population, have been subjected to intensive rounds of selfing and selection in order to develop two distinct varieties from one population.

**Figure 3:**
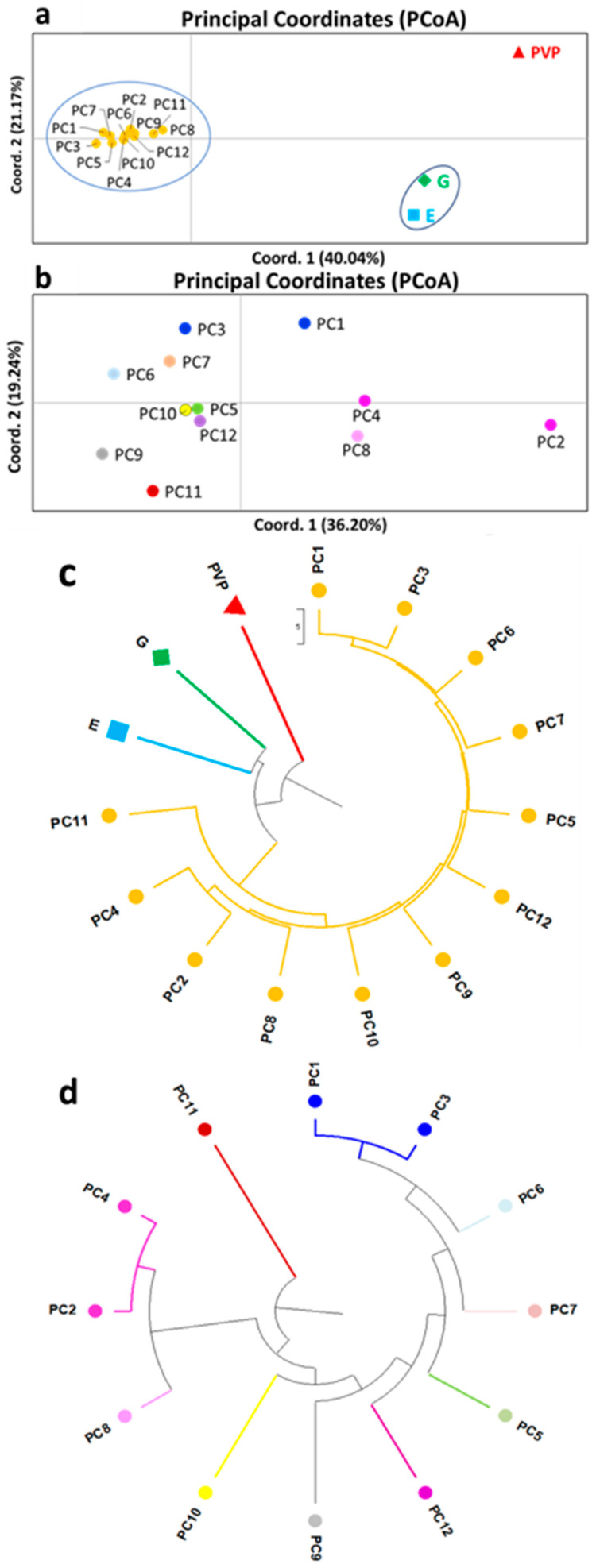
Genetic variation of bean landraces/varieties based on SSR and EST-SSR molecular markers. (**a**) Principal Coordinate Analysis (PCoA) of twelve *P. coccineus* L. landraces (PC1–PC12) compared to the Prespon bean landraces/varieties (“G”, “E”, and “PVP”) and (**b**) PCoA of only the twelve (PC1–PC12) *P. coccineus* L. landraces. (**c**) UPGMA dendrogram of the PC1–PC12 *P. coccineus* L. landraces compared to Prespon bean landraces/varieties (“G”, “E”, and “PVP”), and (**d**) UPGMA dendrogram of the analysis performed on the PC1–PC12 *P. coccineus* L. landraces without the Prespon landraces/varieties.

Figure 3 presents the genetic relationship of the PC1–PC12 Greek landraces of *P. coccineus* L. obtained from the GenBank and originating from other regions in Greece, along with the “G”, “E”, and “PVP” Prespon landraces/varieties (Table 1). The PCoA of the studied bean samples showed a clear distinction of the *P. coccineus* L. landraces PC1–PC12 from those of Prespon landraces/varieties (“G”, “E”, and “PVP”), implying that the PC1–PC12 landraces, are genetically distinct from the Prespon area landraces/varieties. Moreover, the PC1–PC12 landraces exhibited tight clustering along the PCoA coordinates, indicating a rather high genetic similarity (Figure 3a). To further distinguish the genetic diversity among the PC1–PC12 landraces, the PCoA was exclusively carried out for this group of samples on their own, after removing the Prespon landraces/varieties from the analysis (Figure 3c).

**Figure 4:**
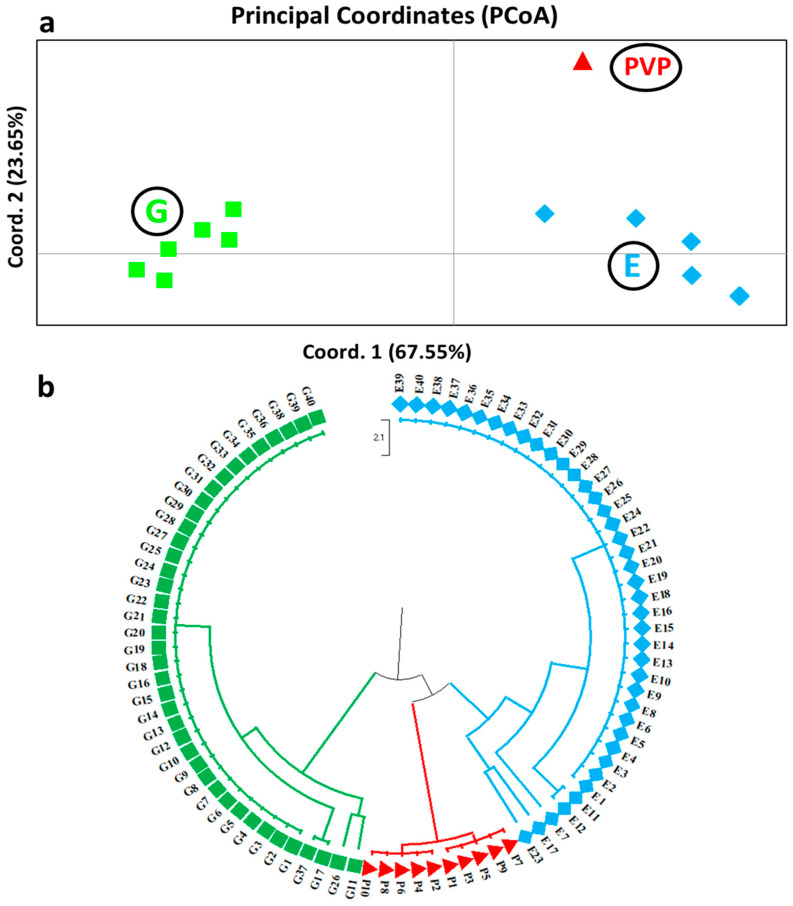
Genetic analysis of the Prespon bean landraces/varieties using SSR and EST-SSR molecular markers. (**a**) Principal Coordinates Analysis (PCoA) and (**b**) UPGMA dendrogram of the three studied Phaseolus landraces/varieties groups, namely “Gigantes” Prespon (green), “Elephantes” Prespon (blue), and Plake Megalosperma Prespon, “PVP” (red). The individuals of the landraces/varieties clustered into the three groups are highlighted in different colors.

The PCoA further confirmed the genetic link. The population distance matrix was utilized as input for panel A (PCoA for *P. coccineus* L. landraces and a *P. vulgaris* L. variety), and coordinates 1 and 2 explained 40.04% and 21.17% of the variance, respectively (Figure 3a). The PCoA of the 12 *P. coccineus* L. landraces, after excluding the Prespon landraces/varieties samples, accounted for coordinates 1 and 2, describing 36.20% and 19.24% of the overall variation, respectively (Figure 3b). On the other hand, the genetic distance data of panel C, performed for the Prespon landraces/varieties (PCoA), revealed that coordinates 1 and 2 explained 67.55% and 23.65% of the differences in variability, respectively (Figure 4a).

To further examine landrace clustering, UPGMA dendrograms were also generated for both analyses, i.e., with or without the inclusion of the Prespon bean landraces/varieties (“G”, “E”, and “PVP”) (Figure 3c,d). In Figure 3c, the formation of three clusters is noted. The first cluster consists of “G” and “E” Prespon landraces; the second includes only the “PVP” Prespon variety, and the third comprises the 12 *P. coccineus* L. landraces (PC1–PC12). The latter analysis could also segregate the “G”, “E”, and “PVP” landraces/varieties, as occurred with the PCoA analysis. However, another UPGMA dendrogram was exclusively carried out for only PC1–PC12 landraces after removing the Prespon landraces/varieties (“G”, “E,” and “PVP”) (Figure 3d) for more clarity. The UPGMA dendrogram of the PC1–PC12 landraces (Figure 3d) revealed the formation of ten main clusters. The Red-Gigantes Chania-Voutas (PC1), Red-Gigantes Drama-Peliti (PC3), and Gigantes Kastoria-Melas (PC6) landraces were included in the first cluster. The genotypes within this cluster were further divided into two subclusters, the first of which involved Red-Gigantes Chania-Voutas (PC1) and Red-Gigantes Drama-Peliti (PC3) and the second included Gigantes Kastoria-Melas (PC6). This was expected given that they were the only of the 15 landraces that belonged to the dry red beans, while the rest of them were white beans. Because of this, they did not possess enough genetic differences among themselves, which would allow them to be further distinguished at a molecular level. The following six landraces, namely Elephantes Kastoria-Ano Melas (PC7), Gigantes Kastoria-Korestia (PC5), Gigantes Trikala-Xrysomhlia (PC12), Gigantes Grevena-Monaxiti (PC9), Gigantes Grevena-Spileo (PC10), and Gigantes Arkadia (PC11) formed a clear cluster each, suggesting that they had a distinct genetic identity, i.e., these *P. coccineus* L. landraces could easily be distinguished from the remaining *P. coccineus* L. landraces. The last cluster consisted of Gigantes Fokida-Artotina (PC2), Gigantes Agios Germanos (PC4), and Gigantes Lechovo (PC8), being divided, however, into two subclusters of which the first involved Gigantes Fokida-Artotina (PC2) and Gigantes Agios Germanos (PC4), and the second was Gigantes Lechovo (PC8).

To examine the genetic variation existing amongst the Prespon landraces/varieties (“G”, “E”, and “PVP”), PCoA was also performed only for these samples (Table 2, Figure 4). The three Prespon landraces/varieties were clearly distinguished from each other according to the PCoA analysis (Figure 4a). The expected discrimination of the “PVP” variety, which belongs to the *P. vulgaris* L. species, from the “G” and “E” *P. coccineus* L. landraces, was further supported by the PCoA analysis showing a clear separation amongst the three groups (Figure 4a). Nevertheless, even though the UPGMA dendrogram shows that “E” and “PVP” Prespon landrace/variety are linked, which could have been brought about via agronomic practices (plant cultivation practices permitting intercrossing) and the inter-transfer of genetic material, these two differed genetically (Figure 4b).

The breakup of genetic variation among and within the Phaseolus landraces/varieties was further evaluated by an analysis of molecular variance (AMOVA). (Table 6). The AMOVA results showed that 65% of the total genetic variance was found among accessions and 35% within landraces in the first group of 14 *P. coccineus* L. landraces and the “PVP” variety (a). In the case of (b), after removing Prespon landraces/varieties, the AMOVA results indicated that the major proportion of variance (67%) occurred within the twelve *P. coccineus* L. landraces (PC1–PC12) and the minor proportion (33%) occurred among them. In the last group (c), which included only the Prespon landraces/varieties, the AMOVA analysis indicated that the highest proportion (93%) of the total genetic variance was detected among accessions and the remaining proportion (7%) occurred within them.

### 3.2. Model-Based Clustering Analyses

**Figure 5:**
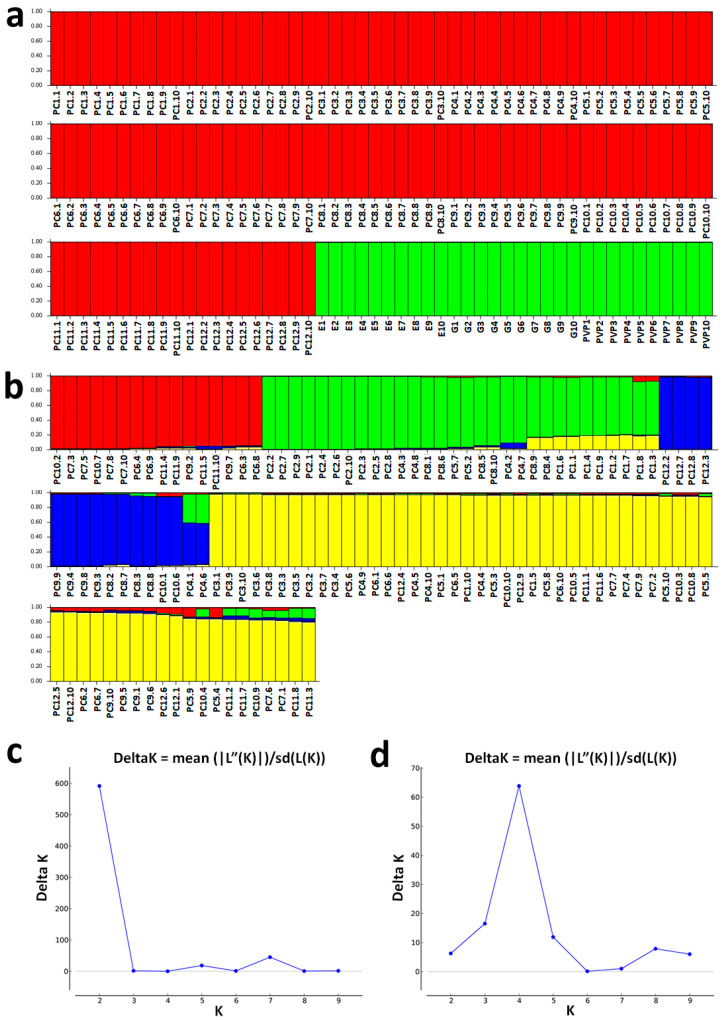
Population structure analysis of bean landraces/varieties based on SSR and EST-SSR data using the STRUCTURE software: (**a**,**c**) Analysis of individuals from twelve *P. coccineus* L. landraces, two *P. coccineus* L. Prespon landraces, and one *P. vulgaris* L. Prespon variety, and (**b**,**d**) Analysis of individuals from twelve *P. coccineus* L. landraces (without inclusion of the Prespon landraces/varieties). The different colors of the bar indicate the four groups identified through the STRUCTURE program. Samples with the identical color exhibit genetic similarity. The corresponding membership probability is presented in the vertical axis. Estimation of the optimum number of mentioned clusters was made according to Evanno et al.’s [30] method. The graphs on the bottom represent the ΔK for each K value. The sharp peak of ΔK at K = 2 suggests the presence of two subgroups in the case of (**a**), and at K = 4, the presence of four subgroups in the second case (**b**).

To further assess the genetic structure of the *P. coccineus* L. landraces (12 landraces from other regions in the country) compared to the Prespon landraces/varieties (“G”, “E”, and “PVP”), population genetic structure analysis was also carried out using the STRUCTURE software. The analysis also showed clear structural discrimination between the Prespon landraces/varieties and the 12 *P. coccineus* L. landraces (PC1–PC12). According to the results of Evanno’s et al. [30] test, two subgroups (K = 2) were the most informative (Figure 5a,c), which is in agreement with the PCoA analysis (Figure 3a,b). The studied landrace’s/variety’s genetic profile was dominated by the group depicted in red, followed by the group illustrated in green. The former mainly included all *P. coccineus* L. landraces (PC1–PC12), while the latter included the Prespon landraces/varieties (“G”, “E”, and “PVP”).

The removal of the Prespon landraces/varieties from the structural analysis revealed four subgroups (K = 4) within the *P. coccineus* L. landraces, as shown by Evanno’s test (Figure 5b,d), which was not clearly shown in the PCoA analysis (Figure 4b). However, after removing the Prespon landraces/varieties (“G”, “E”, and “PVP”) from the structural analysis dataset, it was observed that the remaining landraces were categorized into four subgroups (Figure 5b).

**Figure 6:**
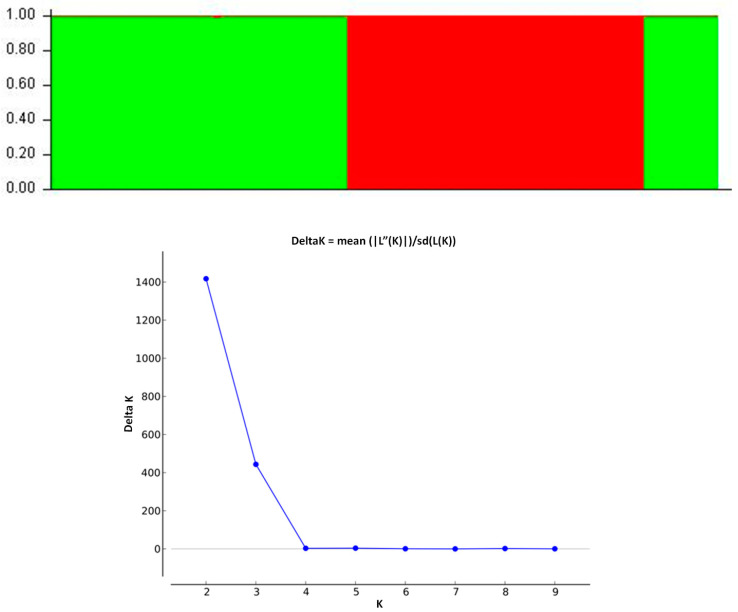
Population structure analysis of Prespon bean landraces/varieties based on SSR and EST-SSR data using the STRUCTURE software. The analysis was performed on the SSR data of four Prespon *P. coccineus* L. genotypes, involving 10 individuals each, and individuals of a *P. vulgaris* L. genotype used as an outlier. Samples with the identical color exhibit genetic similarity. In the vertical axis, the relevant membership probability is displayed; K = 2 was determined according to Evanno’ et al. [30] test.

The analysis of the Prespon landraces/varieties “G”, “E”, and “PVP” with the STRUCTURE software resulted in two subgroups (K = 2) according to Evanno’s test (Figure 6), which is in agreement with the UPGMA dendrogram that showed a distinct separation of the three groups. Nevertheless, in the UPGMA dendrogram (Figure 4b), the “PVP”’ clade was more similar to the “E” clade instead of the “G” clade amongst the Prespon landraces/varieties sample. In conclusion, despite the closer genetic similarity of the “PVP” and “E” Prespon landrace/variety, the “G” Prespon landrace was well separated from the “E” Prespon landrace, which was the main objective of the present study.

## 4. Discussion

Landraces provide a useful gene pool for plant breeding. They frequently encompass an abundance of genetic diversity, which is crucial for the continued viability of conventional agriculture in view of the climatic change [32]. *Phaseolus* spp. genetic improvement and the introduction of new cultivars depend on the effective conservation and usage of this gene pool’s genetic diversity.

The assessment of genetic diversity by molecular analysis can offer new and accurate information that is independent of the environmental impacts [33,34]. The genetic variability of the *Phaseolus* species has been investigated in the past by several researchers, including Rodriguez et al. [17] who analyzed Mesoamerican and European domesticated accessions, as well as wild accessions of *P. coccineus* L. They reported similar heterozygosity (He = 0.29) with other similar studies for the European accessions, detecting the lowest diversity (He = 0.26) in central-northern Europe among the samples they analyzed. Spataro et al. [11] studied wild types and landraces from Mesoamerica and Europe and determined that European accessions have lower genetic variability (He = 0.36–0.43) than the Mesoamerican genetic materials (He = 0.50–0.54), whereas Guerra-García et al. [35] also analyzed Mexican populations belonging to *P. coccineus* L. and *P. vulgaris* L. In the later study, the wild-type group revealed the highest diversity and the cultivated group the lowest, while *P. coccineus* L. showed the highest diversity among the two species [35]. Sicard et al. [10] used inter simple sequence repeat (ISSR), SSR, and chloroplast simple sequence repeat (CpSSR) markers to explore the genetic diversity of *Phaseolus* genotypes from Italy. The authors in this study reported similar (H = 0.27) genetic diversity in some Italian *P. coccineus* L. populations with other studies regarding the European *P. coccineus* L. landraces using SSR molecular markers. Furthermore, SSR microsatellite markers were utilized by Zhang et al. [36] and Desiderio et al. [37] to assess a collection of Chinese and Mesoamerican, respectively, common bean (*P. vulgaris* L.) accessions. Kwak and Gepts [38] also investigated wild and domesticated accessions from Latin America, Europe, the USA, Africa, and Asia from the *Phaseolus vulgaris* World Collection at CIAT (The International Center for Tropical Agriculture, Cali, Colombia) using SSR markers.

Nevertheless, all of the aforementioned studies did not include any Greek bean landraces. In the present study, we examined the genetic diversity and population structure of 14 Greek *P. coccineus* L. landraces and a variety of *P. vulgaris* L. Furthermore, the present work is the second study to appear in the literature for assessing Greek bean landraces. More specifically, this study is focused on how the Prespon “Gigantes” and “Elephantes” landraces differ from other Greek bean landraces, as well as on intraspecific and interspecific genetic diversity, offering valuable information for future breeding programs and a genetic framework analysis to distinguish this specific groups of *P. coccineus* L. landraces for authenticity purposes. Previous works on the genetic diversity of beans have also demonstrated the successful separation of various genotypes into distinct groups [39,40].

The landraces/varieties analyzed herein showed relatively low levels of genetic diversity (mean He = 0.304), while in other studies investigating *P. coccineus* L. wild and/or domesticated landraces [12,16,17,35], even lower values have been reported. Notably, in the studies of Spataro et al. (2011) [11], higher levels of heterozygosity were measured amongst several Mesoamerican wild genotypes and landraces. This result indicates that the analyzed Greek *P. coccineus* L. landraces are among the European populations with the highest genetic diversity, in comparison to the relevant findings of Spataro et al. [11], Mercati et al. [12], and Desiderio et al. [37]. Moreover, the AMOVA analysis revealed that there was more genetic diversity among the studied landraces (65%) than within them (35%). Concerning the Prespon landrace/variety sub-group, the respective percentages were 93% among and only 7% within the landraces/varieties. However, when considering only the *P. coccineus* L. landraces without the Prespon sub-group, the genetic diversity among them (33%) was greatly reduced in relation to the genetic diversity within them (67%). These results indicate that there is a higher genetic variation amongst the Prespon landraces/varieties in comparison to the rest of the Greek *P. coccineus* L. landraces, which appeared to be less genetically diverse. Furthermore, looking at the geographical map, it also appears that some *P. coccineus* L. landraces likely originated from nearby regions, and this fact may underlie the reduction in heterogeneity.

The PCoA and UPGMA analyses revealed that the Prespon landraces/varieties populations (“G”, “E”, and “PVP”) formed separate clusters from the rest of the *P. coccineus* L. landraces (PC1–PC12), implying that there is distinct genetic variation in the Prespon landraces, probably due to their geographic isolation in the Prespes Lake area. Significant separation among the PC1–PC12 samples was only possible when removing the Prespon group from the analysis, indicating that the presence of the latter group ‘masks’ any variation encountered within the PC1–PC12 landraces. This result was further supported by the STRUCTURE analysis of all the analyzed bean landraces (PC1–PC12, “G”, “E”, and “PVP”). More specifically, this analysis showed that the different Prespon varieties have very similar genetic material composition, especially when compared to that of the other Greek landraces. Although accessions from two distinct *Phaseolus* species (*P. coccineus* L. and *P. vulgaris* L.) were present in the Prespon group, they seemed to be more genetically similar than with the respective landraces of *P. coccineus* L. from other Greek locations. This is likely the result of outcrossing that has been previously shown to occur between the two species [41]. Therefore, owing to the geographic proximity and local agronomic practices, it is not unlikely for gene flow to occur amongst the Prespon plants than with the more isolated PC1–PC12 landraces. Similar genetic differentiation between geographically separated *P. coccineus* L. landraces has been previously reported amongst European and Mesoamerican collections [11]. In fact, the Mesoamerican *P. coccineus* L. gene pool was more genetically similar to *P. dumosus* from the same region, than to the European *P. coccineus* L. gene pool [11]. Furthermore, the relatively low genetic diversity and high structure of the examined landraces examined in the present study might be the random and limited distribution range, as well as the small sizes of landraces along with the selfing and inbreeding, potentially leading to low genetic diversity.

Removing the Prespon landraces/varieties and performing two separate structure analyses, one including only the Prespon group and the other only the rest of the samples, distinct subgroups were identified. Four groups were formed within the PC1–PC12 subgroup, while two groups were revealed in the Prespon landraces/varieties. In the Prespon group, the “G” is clearly distinct from both the “E” and “PVP” samples, which seem to be the most genetically similar groups based on all three types of analysis. Possibly, by adding more markers, the genetic differentiation between the “E” and “PVP” Prespon landrace/variety could become even more apparent.

Overall, the genetic analyses performed in the present study have the potential to discriminate and characterize bean landraces and varieties. Independent of environmental impacts, molecular analysis can provide new information useful for ample characterization of the genetic material and the origin of crops [42]. Furthermore, molecular analysis is anticipated to make contributions, along with other innovative methodologies that combine information from DNA sequences with desirable nutritional qualities and sensory attributes, to the evolving fields in human nutrition of nutrigenomics and molecular gastronomy [43]. The present study clearly demonstrated that the establishment of DNA fingerprinting for bean landraces planted in the region of the Prespes Lake area, Greece, will be helpful for planting, selecting, and producing bean cultivars.

## 5. Conclusions

Assessing the genetic diversity of germplasm, a process also referred to as prebreeding, constitutes the first phase in the process of developing cultivars of improved quality attributes. To safeguard the bean’s genetic resources, it is crucial to gather and assess many local landraces for their genetic diversity. The present study focused on the investigation of the intra- and inter-genetic variability among the Greek *P. coccineus* L. plant resources, including the Prespon landraces (“G” and “E”). The research findings revealed that a significant degree of genetic diversity exists within the Greek bean landraces, especially for the *P. coccineus* L. Prespon gene pool, which was clearly distinct from other Greek landraces of this species. This study also demonstrated that the genetic diversity among *Phaseolus* landraces can be effectively determined by employing SSR and EST-SSR marker analyses. These results are expected to assist in the choice of suitable markers for further investigations into the studies of bean genetic variation. In this context, molecular characterization and microsatellite genotyping of the rich Greek bean germplasm using appropriate molecular markers can further aid in the genetic identification of “G” and “E” Prespon beans, thus preventing any form of adulteration in marketing this distinct group of cultivated beans as certified products of Protected Geographical Indication. The results acquired can also be useful for the management and sustainable genetic conservation of *Phaseolus* landraces, particularly those that come from the Prespon area. Further molecular research on national collections of cultivated plants, which include local varieties, landraces, hybrids, introduced accessions, breeding lines, and even wild species, may be required to gain a better understanding of the gene pool of Prespon landraces and varieties and its unique genetic identity in comparison to other extant local varieties and landraces of Greece.

## Figures and Tables

**Table 1:** Local landraces of *P. coccineus* L. (PC, E, G) and one variety of *P. vulgaris* L. (PVP) used in this study.

	Code	Runner Bean	Species	Local Name *
1	PC1	Red Gigantes	*P. coccineus* L.	Chania-Voutas
2	PC2	Gigantes	*P. coccineus* L.	Fokida-Artotina
3	PC3	Red Gigantes	*P. coccineus* L.	Drama-Peliti
4	PC4	Gigantes	*P. coccineus* L.	Agios Germanos
5	PC5	Gigantes	*P. coccineus* L.	Kastoria-Korestia
6	PC6	Gigantes	*P. coccineus* L.	Kastoria-Melas
7	PC7	Elephantes	*P. coccineus* L.	Kastoria-Ano Melas
8	PC8	Gigantes	*P. coccineus* L.	Lechovo
9	PC9	Gigantes	*P. coccineus* L.	Grevena-Monaxiti
10	PC10	Gigantes	*P. coccineus* L.	Grevena-Spileo
11	PC11	Gigantes	*P. coccineus* L.	Arkadia-Kato Dadia
12	PC12	Gigantes	*P. coccineus* L.	Trikala-Xrysomhlia
13	E	Elephantes	*P. coccineus* L.	Prespon
14	G	Gigantes	*P. coccineus* L.	Prespon
15	PVP	Plake	*P. vulgaris* L.	Prespon-Laimos

* More information on some of the bean cultivars presented, herein can be found on the website of the Ministry of Rural Development “http://www.minagric.gr/index.php/el/for-farmer-2/crop-production/phixanthi-ospria (accessed on 18 October 2023)”.

**Table 2:** “Gigantes” (G), “Elephantes” (E), and Plake Megalosperma Prespon (P) genotypes used in this study.

	Individual	Genotype	Species	Local Name
1	G1–G10	6G	*P. coccineus* L.	Gigantes Prespon
2	G11–G20	5G	*P. coccineus* L.	
3	G21–G30	13G	*P. coccineus* L.	
4	G31–G40	16G	*P. coccineus* L.	
5	E1–E10	1E	*P. coccineus* L.	Elephantes Prespon
6	E11–E20	4E	*P. coccineus* L.	
7	E21–E30	9E	*P. coccineus* L.	
8	E31–E40	27E	*P. coccineus* L.	
9	P1–P10	Plake	*P. vulgaris* L.	Plake Prespon

**Table 3:** Microsatellite markers (SSR and EST-SSR) utilized for the assessment of genetic diversity in 14 *P. coccineus* L. landraces and one *P. vulgaris* L. variety, as well as in the Prespon landraces/varieties.

Primer Name	Sequence		T_a_ (°C)	Predicted Size (bp)	Molecular Marker	Fluore -Scence Dye	F_st_
AY298744	F	5′-CATAACATCGAAGCCTCACAGT-3′	47	140–175	SSR	ROX	0.518
	R	3′-ACGTGCGTACGAATACTCAGTC-5′					
AZ301561	F	5′-CAGTAAATATTGGCGTGGATGA-3′	47	200–230	SSR	ROX	0.487
	R	3′-TGAAAGTGCAGAGTGGTGGA-5′					
X80051	F	5′-AGTTAAATTATACGAGGTTAGCCTAAATC-3′	49	220–240	SSR	HEX	0.362
	R	3′-CATTCCCTTCACACATTCACCG-5′					
X79722	F	5′-CCAACCACATTCTTCCCTACGTC-3′	49	143–173	SSR	FAM	0.421
	R	3′-GCGGAGGCAGTTATCTTTAGGAGTG-5′					
X04660	F	5′-TTGATGACGTGGATGCATTGC-3′	47	190–220	SSR	HEX	0.857
	R	3′-AAAGGGCTAGGGAGAGTAAGTTGG-5′					
J01263	F	5′-ATGCATGTTCCAACCACCTTCTC-3′	49	220–240	SSR	TAM	0.766
	R	3′-GGAGTGGAACCCTTGCCTCTCATC-5′					
AZ044945	F	5′-CATCAACAAGGACAGCCTCA-3′	47	140–175	SSR	TAM	0.461
	R	3′-GCAGCTGGCGGGTAAAACAG-5′					
endo	F	5′-TCGAGTCACCATATGCCAGA-3′	49	220–250	EST-SSR	FAM	0.138
	R	3′-CAAAGATTGATCCCGAGTGG-5′					
transmemb	F	5′-CAAACCCCAATGACACATGA-3′	48	220–230	EST-SSR	TAM	0.121
	R	3′-TGCTAGAGTGGCTTGGTTCA-5′					
catalase	F	5′-CTTTCCCTGTCGAAGTTTGC-3′	48	220–250	EST-SSR	HEX	0.153
	R	3′-CATCAACCGCCTTCAATTCT-5′					
hyp	F	5′-TGGCTAGTGGTAGCCTTTGG-3′	47	310–320	EST-SSR	FAM	0.825
	R	3′-CTGAACGTGCCTGCAGATAA-5′					
Ser	F	5′-ACGAAATGGAGCTGGGATTA-3′	49	230–250	EST-SSR	TAM	0.139
	R	3′-CCCAGGACTGCACTTCGTAT-5′					
Sial	F	5′-TTTTTGCTTTCAGTGCCAGA-3′	48	220–240	EST-SSR	HEX	0.110
	R	3′-CCAGCTCTCTTGGACCAAAC-5′					
bHLH	F	5′-CCATGACTGGCATCATCATC-3′	49	220–280	EST-SSR	ROX	0.268
	R	3′-GGCCTTTTCTCCAACAACAA-5′					

T_a_: Temperature annealing, F_st_: gene differentiation coefficient.

**Table 4:** Genetic diversity estimates of 14 *P. coccineus* L. landraces and one *P. vulgaris* L. variety.

Landrace/ Variety	N	Na	Ne	Ho	He	P (%)	I	F_st_
PC1	10	1.667	1.456	0.403	0.253	66.67	0.372	0.453
PC2	10	1.667	1.542	0.520	0.292	66.67	0.418	
PC3	10	1.667	1.549	0.520	0.284	60.00	0.407	
PC4	10	2.000	1.685	0.440	0.367	86.67	0.548	
PC5	10	2.067	1.685	0.507	0.348	80.00	0.539	
PC6	10	1.867	1.652	0.480	0.324	73.33	0.485	
PC7	10	1.933	1.691	0.467	0.332	73.33	0.507	
PC8	10	2.133	1.881	0.578	0.391	80.00	0.606	
PC9	10	1.933	1.759	0.587	0.377	80.00	0.556	
PC10	10	2.000	1.752	0.440	0.354	73.33	0.545	
PC11	10	2.133	1.899	0.556	0.407	80.00	0.625	
PC12	10	1.933	1.806	0.483	0.384	80.00	0.567	
E	10	1.400	1.357	0.343	0.186	40.00	0.262	
G	10	1.333	1.323	0.307	0.164	33.33	0.228	
PVP	10	1.000	0.957	0.173	0.103	20.00	0.156	
Mean	10	1.782	1.600	0.454	0.304	66.22	0.455	

N: number of individuals; Na: No. of Different Alleles; Ne: effective number of alleles; Ho: Observed heterozygosity; He: expected heterozygosity; I: Shannon’s diversity index; P (%): percentage of polymorphic loci; F_st_: gene differentiation coefficient.

**Table 5:** Genetic diversity estimates of Prespon landraces/varieties (“G”, “E”, and “PVP”).

Landrace/ Variety	N	Na	Ne	Ho	He	P (%)	I	F_st_
E	10	1.143	1.143	0.143	0.071	14.29	0.099	0.802
G	10	1.429	1.429	0.429	0.214	42.86	0.297	
PVP	10	1.143	1.095	0.143	0.089	14.29	0.149	
Mean	10	1.238	1.222	0.238	0.125	23.81	0.182	

N: number of individuals; Na: No. of Different Alleles; Ne: effective number of alleles; Ho: Observed heterozygosity; He: expected heterozygosity; I: Shannon’s diversity index; P (%): percentage of polymorphic loci; F_st_: gene differentiation coefficient.

**Table 6:** Analysis of molecular variance (AMOVA) of (a) *P. coccineus* L. landraces with “G”, “E”, and “PVP” landraces/varieties, based on 14 microsatellite markers. (b) *P. coccineus* L. landraces after removing the Prespon landraces/varieties, and (c) only “G”, “E”, and “PVP” landraces/varieties.

	df	SS	MS	Est.var	PV	*p*-Value
**(a) SV (*P. coccineus*** **L.)**						
Among Pops	14	1113.573	79.541	7.549	65%	0.607
Within Pops	135	546.400	4.047	4.047	35%	0.001
Total	149	1659.973		11.597	100%	
**(b) SV (*P. coccineus*** **L.)**						
Among Pops	11	317.433	28.858	2.406	33%	0.284
Within Pops	108	518.400	4.800	4.800	67%	0.001
Total	119	835.833		7.206	100%	
**(c) SV (Gigantes-Elephantes-Plake Prespon)**						
Among Pops	2	506.797	253.399	9.477	93%	0.934
Within Pops	87	58.225	0.669	0.669	7%	0.001
Total	89	565.022		10.147	100%	

df: degrees of freedom, SS: sum of squares, MS: mean squares, Est.var: estimated variance component, PV: proportion of variance.

## Data Availability

The datasets used in this study are not publicly available, but can be requested from the corresponding author of the manuscript.
